# Recent Advances in the Extraction of Polycyclic Aromatic Hydrocarbons from Environmental Samples

**DOI:** 10.3390/molecules25092182

**Published:** 2020-05-07

**Authors:** Natalia Manousi, George A. Zachariadis

**Affiliations:** Laboratory of Analytical Chemistry, Department of Chemistry, Aristotle University of Thessaloniki, 54124 Thessaloniki, Greece

**Keywords:** PAHs, sample preparation, environmental samples, extraction, MSPE, SPME, FPSE, SBSE, DSPE, PT-SPE.

## Abstract

Polycyclic aromatic hydrocarbons (PAHs) comprise a group of chemical compounds consisting of two or more fused benzene rings. PAHs exhibit hydrophobicity and low water solubility, while some of their members are toxic substances resistant to degradation. Due to their low levels in environmental matrices, a preconcentration step is usually required for their determination. Nowadays, there is a wide variety of sample preparation techniques, including micro-extraction techniques (e.g., solid-phase microextraction and liquid phase microextraction) and miniaturized extraction techniques (e.g., dispersive solid-phase extraction, magnetic solid-phase extraction, stir bar sorptive extraction, fabric phase sorptive extraction etc.). Compared to the conventional sample preparation techniques, these novel techniques show some benefits, including reduced organic solvent consumption, while they are time and cost efficient. A plethora of adsorbents, such as metal-organic frameworks, carbon-based materials and molecularly imprinted polymers, have been successfully coupled with a wide variety of extraction techniques. This review focuses on the recent advances in the extraction techniques of PAHs from environmental matrices, utilizing novel sample preparation approaches and adsorbents.

## 1. Introduction

**Figure 1:**
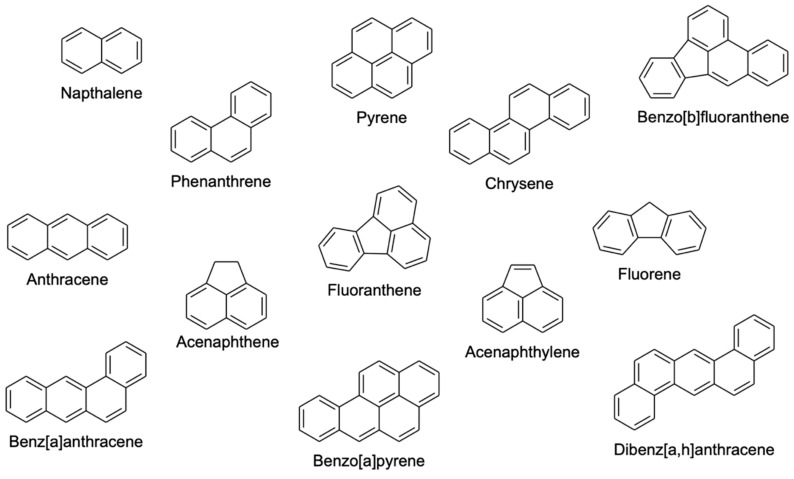
Chemical structures of common polycyclic aromatic hydrocarbons (PAHs)**.**

Polycyclic aromatic hydrocarbons (PAHs) are a large group of chemical compounds composed of two or more fused benzene rings [1]. PAHs are hydrophobic compounds with low water solubility, and their solubility in water and volatility decrease with an increase in their molecular weight [2]. PAHs consisting of up to four rings are known as light PAHs, while PAHs that are made of more than four rings are known as heavy PAHs. Heavy PAHs are more stable and more toxic than the light PAHs. These chemical compounds are widespread environmental contaminants that are considered byproducts of the incomplete combustion of organic materials, such as coal, gas, garbage, meat, oil, tobacco and wood, during natural or anthropogenic processes [1,3]. PAHs are toxic substances, which are resistant to degradation and exposure to them may increase the risk of cancer [4]. As a result, PAHs are considered by US Environmental Protection Agency (EPA) and the European Environmental Agency to be priority pollutants [1]. Therefore, the determination of PAHs in environmental samples is of high importance. Since PAHs exist in traces in environmental matrices, a preconcentration technique is normally required. Figure 1 shows the chemical structures of common PAHs.

Currently, the most widely used methods for analyzing these pollutants in environmental matrices are gas chromatography (GC), high performance liquid chromatography (HPLC) and ultra-high pressure liquid chromatography (UHPLC) [3,5]. Various detection systems, including ultraviolet detectors (UV) [6], diode array detectors (DAD) [3], fluorescence detectors (FLD) [1], mass detectors (MS) coupled with HPLC and UHPLC and flame ionization detectors (FID) [7], MS detectors [5] and tandem MS detectors (MS/MS) [8] coupled with GC have been used. Due to the enhanced sensitivity in the determination of PAHs that results in lower LODs, mass detectors and tandem MS detectors are generally preferred.

Solid-phase extraction (SPE) and liquid–liquid extraction (LLE) are two major sample preparation techniques that have been widely used for the extraction and preconcentration of a wide variety of analytes from environmental samples. However, both conventional techniques tend to have many fundamental drawbacks, since they include complicated, time-consuming steps, while they require large amounts of sample and organic solvents. Moreover, in both techniques there are difficulties in automation [9,10].

In order to overcome these drawbacks, different microextraction techniques have been proposed as an efficient alternative to classical extraction techniques, since the introduction of solid-phase microextraction (SPME) by the research group of Pawliszyn [11]. Liquid-phase microextraction (LPME) was introduced a few years later by Liu and Dasgupta, by using organic droplets suspended from the tip of a microsyringe [12]. Those microextraction techniques are widely used today, and they offer certain benefits compared to the conventional sample preparation techniques. Microextraction techniques require a significantly lower sample amount, number of extraction steps, sample preparation time and organic solvent consumption, and they comply with Green Analytical Chemistry principles [10,13].

Typical examples of miniaturized sample preparation techniques include dispersive solid phase extraction (d-SPE) [14], magnetic solid-phase extraction (MSPE) [15], pipette tip solid-phase extraction (PT-SPE) [16], fabric phase sorptive extraction (FPSE) [17], stir bar sorptive extraction (SBSE) [18] etc. In recent years, a wide variety of novel sorbents, including molecular imprinted polymers (MIPs), graphene, graphene oxide (GO), carbon nanotubes (CNTs), metal organic frameworks (MOFs), covalent organic frameworks (COFs) and zeolitic imidazole frameworks (ZIFs) have been successfully coupled with miniaturized extraction techniques and microextraction techniques [14,19,20,21].

Until now, a plethora of publications discuss the different analytical procedures for the extraction of PAHs from liquid, solid and air matrices [22,23,24,25,26,27,28,29]. To the best of our knowledge, in the last five years there were no review papers regarding the extraction techniques of PAHs from environmental samples with the novel miniaturized extraction and microextraction techniques. Herein, we aim to discuss the recent advances in the extraction of PAHs from environmental samples. Emphasis will be given to the miniaturized sample preparation approaches, as well as the novel sorbents and other materials that have been successfully coupled with various microextraction techniques.

## 2. Extraction of PAHs from Environmental Matrices

**Figure 2:**
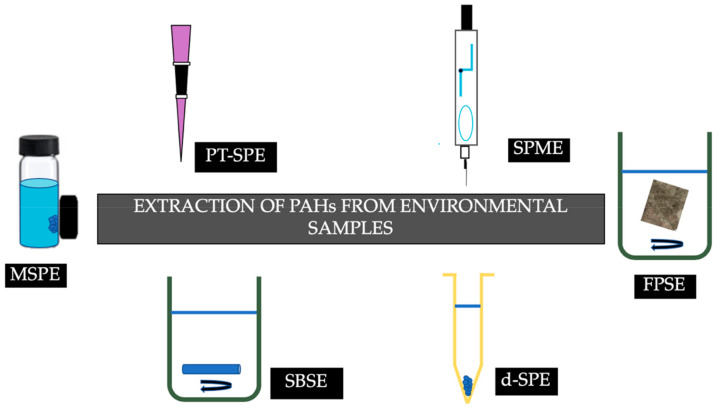
Recent advances in sorptive extraction techniques for the determination of PAHs from environmental samples.

A plethora of novel sample preparation techniques have been employed for the extraction of PAHs. Figure 2 summarizes the recent advances in sorptive extraction techniques that have been employed for the determination of PAHs from environmental samples.

### 2.1. Dispersive Solid-Phase Extraction of PAHs from Environmental Matrices.

Dispersive solid-phase extraction (d-SPE), is a form of SPE in which the desired sorbent is added directly into the sample aqueous solution followed by dispersion. This technique is taking advantage of the contact between the adsorbent and the target analytes. Once the extraction process is completed, the sorbent with the adsorbed analytes is separated from the sample by a mechanical process, such as centrifugation or filtration. Compared to the conventional SPE process, the main benefit of d-SPE is the reduction of sample preparation time, as well as its simplicity, adaptability and easy handling. A wide variety of sorbents have been utilized for the d-SPE of PAHs from environmental samples [30,31].

This technique gained popularity when Anastassiades et al. [32] introduced the QuEChERS (Quick, Easy, Cheap, Effective, Rugged and Safe) approach for the determination of pesticide residue in food of plant origin. The initial method consists of acetonitrile extraction and addition of a mixture of salts, followed by a dispersive clean-up step with a primary–secondary amine (PSA) as extraction sorbent. QuEChERS was quickly applied for the determination of other analytes in a variety of sample matrices. Cvetkovic et al. [33] developed a QuEChERS extraction procedure of PAHs in soil prior to their determination by GC-MS. The researchers evaluated different solvent systems (acetonitrile/water and hexane/water) and sorbents (PSA, C_18_, Florisil, diatomaceous earth and clinoptilolite). Among the tested parameters, the best results were obtained with acetonitrile/water, as the extraction solvent and diatomaceous earth as the d-SPE extraction adsorbent.

Until today, a wide variety of novel extraction sorbents have been evaluated for the d-SPE of PAHs from environmental matrices. Among them, metal-organic frameworks and zeolitic imidazole frameworks are currently the most popular d-SPE adsorbents. MOFs became widely known in 1995, when Yaghi and Li [34] reported the hydrothermal synthesis of a MOF material with large rectangular channels. Metal-organic frameworks are a class of hybrid organic-inorganic supramolecular materials, which are based on the coordination of metal ions or clusters with bi- or multidentate organic linkers. What makes MOFs materials so attractive is their unique properties, such as high surface areas (up to 14,600 m^2^·g^−1^) [35], pore size tunability, structure flexibility, luminosity, thermal stability, charge transfer ability from the ligand to the metal or from the metal to the ligands, etc [21,36,37,38,39]. As a result, MOFs have gained attention in a plethora of applications, such as gas storage and separation [40], catalysis [41], sensors [42], detoxification [43] and drug delivery [44]. In analytical chemistry, MOFs have been evaluated as stationary phases for GC [45,46] and HPLC [47,48] analysis. However, today, the most popular field of applications of MOFs in analytical chemistry is sample preparation [21,36].

Xia et al. [49] synthesized a JUC-48 (Jilin University China 48) from cadmium nitrate tetrahydrate and biphenyl-4,4’-dicarboxylic acid, and used it for the d-SPE of PAHs from environmental matrices prior to their determination by HPLC-FLD. The novel sorbent exhibited good characteristics regarding its stability and morphology, high surface area and open adsorption site. The researchers observed highly negative relationship between extraction capacity and molecule size. As a result, the JUC-48 adsorbent was used for the extraction of only light PAHs.

Amiri et al. [50] synthesized hybrid nanocomposites prepared from MOF-199 and graphene or fullerene. MOF-199 was prepared from copper nitrate trihydrate and 1,3,5-benzenetricarboxylic acid. Among the examined nanocomposites (i.e., MOF-199, MOF-199/graphene and MOF-199/fullerene), the MOF-199/graphene nanocomposite exhibited the highest adsorption affinity towards PAHs, probably due to the high porosity, surface area and adsorption capacity of graphene. 

Zeolitic imidazole frameworks (ZIFs) are a sub-family of metal organic frameworks that favors the benefits of both zeolites and MOFs. ZIFs are composed of imidazolate linkers and metal ions. Their structures are similar to conventional aluminosilicate zeolites. Due to their intrinsic porous characteristics and abundant functionalities, as well as exceptional thermal and chemical stabilities, ZIFs have a wide range of potential applications [37,51].

Liang et al. [52] evaluated the application of a novel cellulose/zeolitic imidazolate frameworks-8 composite for the d-SPE of PAHs from environmental water samples. ZIF-8 is composed of zinc as metal ion and 2-methyl imidazole as an organic linker, and it exhibits good characteristics for the extraction of PAHs due to strong hydrophobic and π–π interaction, as well as permanent porosity and high surface area. However, ZIF-8 suffers from tiny inner pores and strong hydrophobicity. In order to overcome this drawback, ZIF-8 was modified with cellulose microspheres (CMs) generated from natural cellulose. CMs exhibit macro/mesoporous structures, high surface area and abundance of hydroxyl groups. Due to the combination of the benefits of both ZIF-8 and the CMs, the hybrid material exhibited good extraction characteristics.

Other d-SPE sorbents that have been applied for the extraction of PAHs from environmental samples include graphene/sepiolite [53] and *N*-acetyl-l-cysteine modified CdS quantum dots [54].

### 2.2. Magnetic Solid-Phase Extraction of PAHs from Environmental Matrices.

Magnetic solid-phase extraction (MSPE) is a form of d-SPE in which a magnetic nanomaterial is added into an aqueous sample solution to adsorb the target analytes. After the adsorption of the analyte, an external magnetic field is applied to collect the sorbent and the supernatant solution is discarded. Subsequently, elution of the adsorbed analytes is achieved with the addition of an appropriate solvent, and magnetic separation is performed once again to collect the eluent, which is further analyzed by a suitable analytical technique. Compared to the conventional SPE procedure, in MSPE there is no need for sorbent packing into cartridges, thus avoiding limitations of column blocking and high pressure. Meanwhile, sample and organic solvent consumption is significantly decreased compared to the classic SPE and LLE formats. Finally, the sorbent separation with a magnet is a simple and rapid process, compared to the time-consuming centrifugation and filtration steps that are required in conventional d-SPE [55,56,57].

Magnetic nanoparticles (MNPs) are characterized by the general formula MFe_2_O_4_ (M = Fe, Co, Cu, Mn, etc.), and they can be produced by a variety of methods, such as co-precipitation, solvothermal, hydrothermal etc. The most common magnetic nanoparticles that have been used in order to fabricate magnetic sorbents for MSPE are Fe_3_O_4_ nanoparticles. Iron oxides have been widely used in MSPE due to their super paramagnetism, their low toxicity, their high magnetic saturation, their simple preparation process and their low price [7]. The application of other magnetic nanoparticles, such as MnFe_2_O_4_, have been also reported [58]. However, the utilization of MNPs in sample preparation has some drawbacks, since their selectivity is low. Moreover, MNPs exhibit low stability in strong acidic aqueous media and low dispersion ability in many sample matrices. Therefore, surface modification of magnetic nanoparticles is usually required to enhance their stability and selectivity by the introduction of special functional groups [7]. As seen in Table 1, a wide variety of chemical compounds including carbon-based materials, polymeric materials, MOFs and other molecules have been employed for this purpose.

#### 2.2.1. Metal-Organic Framework Magnetic Nanocomposites for the MSPE of PAHs

A wide variety of metal-organic frameworks have been evaluated as sorbents for the MSPE of PAHs from water samples, after functionalization with magnetic nanoparticles. HKUST-1 (Hong Kong University of Science and Technology), was prepared from copper (II) nitrate hemipentahydrate and benzene-1,3,5-tricarboxylic acid, and has been applied for the MSPE of PAHs from water and fruit tea infusion samples, prior to their determination by UHPLC-FLD. The proposed method provided adequate quantitation and reproducibility of heavy PAHs; however, extraction efficiencies were relatively low and no sorbent reusability was reported [59].

MIL-101(Cr) is another MOF that has been used for the extraction of PAHs after its in situ magnetization [60]. MIL-101 was prepared from chromium nitrate nonahydrate and terephthalic acid, and the magnetic nanocomposite was prepared by the in-situ magnetization of MIL-101 and silica-coated magnetite nanoparticles (Fe_3_O_4_@SiO_2_). The novel sorbent exhibited good extraction characteristics, as well as a low sorbent quantity requirement. MIL-101 (Cr) modified zero valent iron nano-particles have been also evaluated as an MSPE adsorbent for the extraction of PAHs [61]. Nanoscale zero valent iron exhibits increased specific surface area and more reactive sites on the surface, compared to Fe_3_O_4_ nanoparticles. Moreover, it is very easy to prepare core-shell Fe@SiO_2_ through a one-step approach under normal temperature and pressure, which directly provides the SiO_2_ layer. The novel nanocomposite exhibited high extraction efficiency and stability, since it was found to be reusable at least 10 times.

MIL-100(Fe) has been also evaluated as a MSPE sorbent for the extraction of PAHs from water samples [62]. The MIL-100(Fe) sorbent was prepared form iron (III) chloride hexahydrate and 1,3,5-benzenetricarboxylic acid, and it was functionalized with mercaptoacetic acid modified magnetite nanoparticles. The novel sorbent was successfully applied for the MSPE of PAHs from river, well, pond and tap water samples. Although satisfactory extraction recoveries were reported (>80%), no data regarding sorbent reusability were provided. Huo et al. [63] prepared a magnetic MIL-100(Fe) nanocomposite through an pyrolytic in situ magnetization approach. In this case, MIL-100 (Fe) was placed in a tube furnace and it was calcined from 300 °C to 700 °C under nitrogen. The obtained material exhibited high porosity and magnetic characteristics and it was found to be recyclable and reusable (up to 10 times).

Zhang et al. [64] prepared a zinc benzimidazolate (ZIF-7) nanomaterial anchored onto polydopamine-coated Fe_3_O_4_ nanoparticles. Due to the hydrophobic and π−π interaction between ZIF and the target analytes, good extraction efficiency was reported. Moreover, the novel nanocomposite was found to be reusable for at least 10 times.

Covalent organic frameworks (COFs) are novel materials, structurally related to MOFs, consisting of light elements (H, O, C, N, B, Si) connected with organic monomers through strong covalent bonds. COFs comprise a class of ordered crystalline porous polymers that are characterized from superior properties, including low crystal density, high specific surface area, pore size tunability and good thermal stability [36,94,95]. He et al. [65] fabricated a bouquet-shaped magnetic porous nanocomposite via grafting a COF prepared from 1,3,5-triformylphloroglucinol and p-phenylenediamine (TpPa-1) onto the surface-modified magnetite nanoparticles. The novel sorbent contained clusters of core−shell magnetic nanoparticles and interconnected porous COF nanofibers. Therefore, it exhibited high specific surface area, high porosity, and supermagnetism.

Another covalent organic framework that has been evaluated as adsorbent for PAHs was COF-LZU1 (= Lan Zhou University-1), after immobilization onto polyethyleneimine-functionalized magnetic nanoparticles (COF-LZU1@PEI@Fe_3_O_4_) [66]. For this purpose, the examined COF was prepared from 1,3,5-triformylbenzene and 1,4-diaminobenzene through a Schiff base reaction. The novel sorbent exhibited good stability and reusability. Moreover, due to the strong π-stacking and hydrophobic interaction, the novel sorbent was successfully applied for the MSPE of PAHs from water and soil samples.

#### 2.2.2. Carbon-Based Magnetic Sorbents for the MSPE of PAHs

Magnetic carbon-based nanomaterials exhibit superior extraction characteristics, due to the combination of magnetic nanoparticles with carbon-based materials that pose many interesting properties including good thermal stability and tunable miscibility, as well as good extraction capability [67].

Carbon coated Fe_3_O_4_ nanoparticles were used for the extraction of PAHs from environmental water samples prior to their determination by HPLC-FLD. Due to the large surface area of the nanomaterial and strong adsorption ability of carbon material, high adsorption capacity and extraction efficiency were obtained [68].

A hydrophilic carbon-ferromagnetic nanocomposite was also evaluated for the extraction of PAHs. The sorbent was designed with a hydrophobic sublayer and a hydrophilic surface. As a result, the sorbent exhibited good adsorption efficiency, due to the hydrophobic sublayer of the material, while good compatibility with the aqueous matrices was reported due to its hydrophilic surface [5].

Carbon nanofibers (CNFs) decorated with Fe_3_O_4_ nanoparticles have been also evaluated as adsorbent for the MSPE of PAHs from water samples. Carbon nanofibers are hydrophobic materials with the ability to establish π–π interaction. Moreover, they exhibit high specific area and flexibility, as well as high mechanical strength [7]. Magnetized graphene (G) layers immobilized on CNFs have been also investigated for the MSPE of PAHs. Graphene is a single layer of carbon atoms that was discovered in 2004 by Geim et al. [96]. In graphene, the carbon atoms are densely packed in a honeycomb crystal lattice, which can be viewed as exfoliated ‘‘graphite sheets’’. Graphene exhibits superior properties, such as a high specific surface area, as well as good chemical stability and thermal stability [6]. The combination of graphene and CNFs provided good adsorption of PAHs that can be attributed to hydrophobic and π–π interactions [67]. A graphene/Fe_3_O_4_@polythiophene (G/Fe_3_O_4_@PT) nanocomposite has been also used for the MSPE of PAHs. PT was employed used to enhance the adsorption capacity of magnetic graphene, and also to provide long service life and stability [69].

Graphene oxide is the chemical compound with a form similar to graphene, which consists of one-atom-thick two-dimensional layers of sp^2^-bonded carbon and is rich in oxygen-containing groups including hydroxyl, carboxyl and epoxy groups. Graphene oxide exhibits high hydrophilicity and dispersibility, as well as good thermal and mechanical stability and high surface area. GO interacts with organic molecules through strong π-stacking, hydrophobic interaction and hydrogen bonding [57].

Magnetic graphene oxide has been used for the extraction of PAHs from water samples. The composite sorbent exhibited ease in separation and satisfactory recoveries [6]. Polystyrene (PS) modified magnetic GO (GO-Fe_3_O_4_@PS) was fabricated to enhance the extraction efficiency. Polystyrene is an inexpensive, high porous, environmentally friendly polymer that contains phenyl and alkyl groups [70]. Another polymeric material that has been employed for the functionalization of magnetic GO is poly(pyrrole-co-aniline) in order to increase its efficiency. Both polyaniline (PANI) and polypyrrole (PPy) are promising materials, due to their good chemical stability and ease of synthesis by the chemical and electrochemical method of polymerization. The PANI and PPy copolymer combines the superior properties of the two conducting polymers and its application for the modification of Fe_3_O_4_/GO can result in a highly efficient sorbent [71].

Carbon nanotubes (CNTs) functionalized magnetite nanoparticles have been also employed for the extraction of PAHs from water samples [72]. CNTs are interesting sorbents for volatile and semi-volatile organic compounds due to hydrogen bonding, π-stacking and hydrophobic interactions. CNTs are divided into single-walled (SWCNTs) and multi-walled carbon nanotubes (MWCNTs), based on whether they consist of one or more sealed tube-shaped layers of graphene, respectively. Adsorption of target analytes can take place in their easily accessible walls, as well as in their interstitial sites [97]. MWCNTs functionalized Fe_3_O_4_ nanoparticles showed good extraction efficiency and selectivity towards PAHs [72].

#### 2.2.3. Molecularly Imprinted Polymers Magnetic Nanocomposites for the MSPE of PAHs

Molecularly Imprinted Polymers (MIPs) are synthetic polymeric materials that are composed of imprinted sites complementary to a specific chemical molecule. MIPs show high affinity towards the target analytes with analogous molecular structure, resulting in high extraction ability [55]. Molecular recognition is attributed to a combination of shape and size and hydrogen bonding, as well as electrostatic and hydrophobic interactions. MIPs are stable at a wide pH and temperature ranges and in most organic solvents, while there is normally no requirement for special storage conditions [98,99].

MIPs can be prepared through covalent, non-covalent and semi-covalent approaches, and their preparation is based on the polymerization of a functional monomer and a cross-linker around a template molecule. The template molecule should be able to interact with the functional monomer to develop complexes that further interact with the cross-linker during the polymerization reaction. Subsequently, the template molecule is removed and a MIP with imprinted sites complementary to the molecular structure and the functional groups of the template is created. Normally, after the synthesis of MIPs, extensive washing is required to remove any residual molecules of template. However, even after many washing steps, bleeding of the template has been reported. In order to overcome this drawback, MIPs can be prepared from templates with chemical structure that is analogue to the target molecules [98,100].

Magnetic MIPs combine the benefits of ease in separation of magnetic materials with the high molecular recognition of MIPs. Many researchers reported the synthesis of magnetic MIPs as adsorbents of PAHs from environmental samples.

Villar-Navarro et al. [73] used a commercially available magnetic MIP (mag-MIP) by NanoMyP^®^ (Granada, Spain) that was able to extract the 16 EPA-regulatd PAHs. The examined sorbent exhibited good extraction efficiencies (98.8–100%) for the more lipophilic PAHs, however, for the less lipophilic PAHs, extraction recovery rates were lower (46–60%). Finally, mag-MIP was found to be reusable at least three times. Benedetti et al. [74] managed to improve the extraction efficiencies by implementing a Plackett-Burman experimental design. In this case, all recovery values were higher than 76%.

Azizi et al. [8] reported the synthesis of a MIP sorbent by surface polymerization onto magnetic Fe_3_O_4_@SiO_2_ nanoparticles through reversible addition fragmentation chain transfer (RAFT) polymerization. For this purpose, methacrylic acid and isopropyl acrylamide were used as functional monomers. In this case, extraction recoveries ranged from 4.5% to 97%, thus extraction of “heavy” PAHs was found to be more efficient compared with the extraction of “lighter” PAHs.

#### 2.2.4. Polymer-Modified Magnetic Nanoparticles for the MSPE of PAHs

Polymers are promising materials in separation science, due to the fact that their network can be chemically anchored around the magnetic core, thus providing large π-conjugated structure, hydrophobicity and polar functional groups for adsorption [58]. Various polymers including polydopamine [75], polypyrrole [76], poly(o-toluidine) [58] and polyaniline [77]. Polyaniline and its derivatives are one of the widely used polymers for the extraction of various types of organic compounds, because of their good stability in high temperature, air and different solvents [58].

Polydopamine (PDA) functionalized magnetic nanoparticles have been also employed for the extraction of PAHs. PDA exhibit several significant advantages, including biocompatibility, good dispersibility in water, as well as multifunctional groups (amino and catechol groups) that enhance the extraction efficiency of PAHs due to π–stacking interaction [75].

Polypopyrole [76], due to its hydrophobicity, large π-conjugated structure, hydrogen bonding and ion exchange properties, exhibits good extraction efficiency and selectivity towards PAHs.

Polyaniline-coated magnetite nanoparticles incorporated in alginate beads have also been employed for the MSPE of PAHs in water samples. In this case, the alginate beads assisted in the increase of the surface area for polyaniline coating, and the novel nanocomposite showed satisfactory extraction efficiency towards the PAH analytes, due to the π–π interactions between the polyaniline moieties [77].

Poly(o-toluidine) (PoT) coated MnFe_2_O_4_ magnetic nanoparticles have been also successfully employed as a magnetic adsorbent for the MSPE of PAHs from water samples [58]. Due to the π−π interactions between the active sites of PoT and PAHs, a satisfactory extraction efficiency was reported.

#### 2.2.5. Ionic liquids Modified Magnetic Nanoparticles for the MSPE of PAHs

Ionic liquids (ILs) are a green alternative to conventional organic solvents that have gained a lot of attention lately. ILs are generally composed of bulky, non-symmetrical organic cations (i.e., imidazolium, pyrrolidinium, pyridinium, ammonium, phosphonium etc.) and different inorganic or organic anions (i.e., tetrafluoroborate anions, bromide anions etc.) [101,102,103]. The increased popularity of ILs may be attributed to their extraordinary properties, such as a negligible vapor pressure, good thermal stability, as well as tunable viscosity and miscibility with water and organic solvents. By choosing the cationic or the anionic constituent of ILs, their polarity, hydrophobicity, viscosity and other chemical and physical properties can be tuned, in order to prepare ILs with the desired characteristics. Due to their special structures, ionic liquids also exhibit good extractability for various organic compounds and metal ions [103]. As a result, ionic liquids have been successfully coupled with various extraction techniques for the extraction of PAHs.

Galán-Cano et al. [78] prepared ionic liquid-coated magnetic nanoparticles (IL-MNP) and used them for the MSPE of PAHs from water samples, prior to their determination by GC-MS. For this purpose, methylimidazolium-chloride was used to modify Fe_3_O_4_@SiO_2_ magnetic nanoparticles by a simple metathesis reaction, using potassium hexafluorophosphate as a reagent. Due to the covalent immobilization of the ionic liquid onto the surface of silica coated Fe_3_O_4_ nanoparticles, the developed sorbent exhibited good stability, and it was found to be reusable for up to 10 times.

Cyano-ionic liquid functionalized magnetic nanoparticles (MNP@CN/IL) have been also employed for the MSPE of PAHs from environmental water samples, prior to their determination by HPLC-DAD [79]. For this purpose, 1-benzyl-3-(trimethoxysilylpropyl)imidazolium chloride was chosen as IL, and it was used to modify the cyano-functionalized magnetic nanoparticle. The combination of the cyano group and IL on the surface of the MNPs provided good extraction efficiency, probably due to the combination of π−π and electrostatic interaction. 

Magnetic nanoparticles coated with a polyaniline-di-cationic ionic liquid (MNP-PANI-DICAT) were prepared and used for the MSPE of PAHs in environmental samples [80]. The novel sorbent combined the benefits of PANI and ionic liquids. Due to the π–π interaction between polyaniline shell and di-cationic IL with PAHs compounds, high extraction efficiency was observed. Moreover, the novel MSPE sorbent exhibited satisfactory stability, since it was found to be reusable up to five times.

Liu et al. [81] fabricated a magnetic ionic liquid functionalized methyl orange nanoparticles (Fe_3_O_4_@IL@MO), by self-assembly of the 1-octadecyl-3-methylimidazolium bromide and methyl orange on the surface of Fe_3_O_4_ silica magnetic nanoparticles. The novel sorbent was successfully applied for the MSPE of PAHs from environmental water samples. Due to the presence of benzene rings of methyl orange and the hydrocarbon chains of ILs, the sorbent provided adsorption sites for organic pollutants through π–π and hydrophobic interactions. The silica modified Fe_3_O_4_ nanoparticles were found to be reusable after washing, however, assembling with IL and methyl orange was also required.

#### 2.2.6. Other Magnetic Nanocomposites for the MSPE of PAHs

Naphthyl functionalized magnetic nanoparticles (Fe_3_O_4_@SiO_2_@Nap) have been used for the extraction of PAHs from river waters, prior to their determination by HPLC-FLD. Due to the condensed cyclic structure and the hydrophobic property of naphthyl, the novel sorbent exhibited satisfactory selectivity and extraction efficiency through π−π interactions [1].

Phosphatidylcholine (PC) is a phospholipid consisting of a long double carbon chain, and the zwitterions’ pair headgroup is composed of phosphate and choline. The extraction of PAHs can be assisted, due to the middle hydrocarbon chains that provide adsorption sites due to hydrophobic interactions. Meanwhile, the zwitterions pair headgroups endow the outer surface of the substrate with perfect hydrophilicity and biocompatibility [82].

Another example of composite material exhibiting both hydrophilicity and hydrophobicity is the magnetic nanocomposite prepared from nanoparticles functionalized with divinylbenzene (DVB) and sulfonate moieties (Fe_3_O_4_-DVB-SO_3_^-^). In this case, the hydrophobic DVB moieties were dedicated for extraction, while the hydrophilic sulfonate groups were introduced to enhance dispersion of the magnetic sorbent in the aqueous sample solution [83]. A hydrophilic–hydrophobic magnetic Fe_3_O_4_-doped polymeric nanoparticle (MPNP), prepared from highly charged poly(styrene-divinylbenzene-co-4-vinylbenzenesulfonic acid sodium salt) has been also employed for the determination of PAHs in environmental matrices [3].

Triphenylamine (TPA) has been employed for the functionalization of magnetic nanoparticles (Fe_3_O_4_/SiO_2_/TPA). Due to the strong π–π conjugate effect between the three benzene rings of TPA and PAHs, satisfactory extraction efficiency and selectivity were observed [84].

Other examples of chemical compounds and groups that have been employed for the functionalization of magnetic nanoparticle for the MSPE of PAHs from environmental samples are C_18_ [85], hemimicelles of alkyl (C_10_-C_18_) carboxylates [86], n-octadecylphosphonic acid [87], nylon 6 [88], cetyltrimethylammonium bromide (CTAB) [89], palm fatty acid [90], 1,4,7,10-tetrabenzyl-1,4,7,10-tetraazacyclododecane (TBCD) [91], tetraazacalix[2]arence[2]triazine (TCT) [92] and *n*-hexadecylsilanol-diol (C_16_-HO) [93].

### 2.3. Solid-Phase Microextraction of PAHs from Environmental Matrices.

Solid-phase microextraction is a sample preparation microextraction technique in which the analytes are directly extracted and preconcentrated at the outer coating of a fused-silica fiber [104]. There are two approaches of SPME that can be used to extract analytes, the headspace (HS-SPME), where the fiber is exposed to the gas phase above the sample, and direct immersion (DI-SPME), where the fiber is directly immersed into the sample solution [105]. After the extraction, desorption takes place either thermally in the injection port of a gas chromatograph or by the addition of an organic solvent [104,105].

Until now, there are many different commercial SPME coated fibers, such as polydimethylsiloxane (PDMS), polydimethylsiloxane/divinylbenzene (PDMS/DVB), polyacrylate (PA), carboxen/polydimethylsiloxane(CAR/PDMS) and divinylbenzene/carboxen/polydimethylsiloxane (DVB/CAR/PDMS) [105]. However, most of them more or less have some disadvantages, such as low selectivity, ease of fiber breakage, a short lifetime and swelling in organic solvents. In order to overcome them, various new coatings have been prepared and evaluated [106].

Ionic liquids and polymeric ionic liquids (PILs) have been successfully employed as SPME coatings, due to their simplicity of synthesis and their high tuneability, that enable the preparation of highly selective fibers [107]. PILs are polymers prepared from IL monomers. Compared with conventional ILs, PILs exhibit a number of advantages when used as coatings in SPME. Generally, PILs often have solid nature and good thermal and mechanical strength, while extraction selectivity is similar with ILs. As a result, they have proved to be more stable coatings [107,108]. Various PILs including poly(1-vinyl-3-hexadecylimidazolium) bis[(trifluoromethyl)sulfonyl]imide [109], poly(1-4-vinylbenzyl)-3-hexadecylimidazolium bis[(trifluoromethyl)sulfonyl]imide [110], poly (1-vinyl-3-octylimidazolium) 2-naphthalene-sulfonate [111] and poly(1-(4-vinylbenzyl)-3-hexadecylimidazolium bis[(trifluoromethyl)sulfonyl]imide [112] have been successfully used as coatings used for the SPME of PAHs. In most cases, the PIL was initially prepared and diluted in a volatile solvent (i.e., acetone or chloroform), and a bare fiber (usually made from stainless steel) was immersed in the solution, followed by slow removal and air drying to remove any excess of solvent that may contribute to high background signals in gas chromatography [109,110,112]. However, in situ polymerization of the IL and creation of the SPME coating on the surface of a stainless steel wire has also been reported [111].

Graphene [113,114], graphene oxide [115,116,117], MWCNTs [118,119] and other carbon based materials [120] have also been successfully employed as SPME sorbent coatings, either neat or combined with other materials in order to generate more efficient composites. These materials exhibit high chemical, thermal and mechanical stability, as well as great affinity towards PAHs. Additionally, due to their unique structures and sufficient surface areas, rapid extraction and desorption of the target analytes can be achieved [107]. Various techniques for the preparation of the coated fibers including the chemical bonding [113,115,117], electrophoretic deposition [119] and sol-gel approaches [120] have been also evaluated.

Μetal-organic frameworks (MOFs) are also promising sorbents for SPME coatings [107,121]. A wide variety of MOF materials, such as HKUST-1 [122], MOF-199 [123], ZIF-8 [124], TMU-6 (Tarbiat Modares University) prepared from N^1^,N^4^-bis((pyridin-4-yl)methylene)-benzene-1,4-diamine and 4,4′-oxybisbenzoic acid) [125], MOF-177 prepared from zinc nitrate hexahydrate and 1,3,5-tris(4-carboxyphenyl)-benzene [126], UiO-6 (University of Oslo) prepared from zirconium chloride and terephthalic acid [127], an ytterbium based MOF prepared from aminoisophthalic acid and 2,2’-bipyridine [128], as well as bio-MOF-1 prepared from 4,4’-biphenyl dicarboxylic acid (BPDC), zinc acetate dihydrate and adenine [129].

The main considerations when preparing MOFs for extraction purposes are their stability under extraction conditions, and their ability to establish interactions with the target analytes. Moreover, when fabricating MOFs as coatings for SPME fibers the mechanical and thermal stability should be carefully evaluated. The main procedures that can be used to obtain MOF coatings include physical adhesion, immersion in a suspension of MOF, in situ growth and electrodeposition [107].

A metal-azolate framework 66 (MAF-66) has been also used for the SPME of PAHs from environmental samples. Metal-azolate frameworks are a subfamily of MOFs that have recently gained attention. MAF-66 was prepared from 3-amino-1,2,4-triazole and zinc hydroxide and was used to fabricate a SPME coating by a layer-by-layer deposition method, providing enhancement factors of 123-3108 [130].

Examples of other materials that have been employed to fabricate SPME coatings for the extraction of PAHs include polyaminithiophenol (PATP) with Au coating [131], poly(3,4-ethylenedioxythiophene)@gold nanoparticles[132], crosslinked methyl methacrylate–polyhedral oligomeric silsesquioxane hybrid polymeric coating [106], nanostructured octadecyl silica [133] and polythiophene/carboxylic acid modified multi-walled carbon nanotube composite [134]. In the latter approach, the researchers developed a novel SPME technology, in which the features of heating the sample, cooling the sorbent and extraction under vacuum condition were combined [134].

PAL (Prep And Load solution) SPME Arrow technique [135] has been also evaluated for the extraction of PAHs from environmental samples. This technique is based on the use of a robust stainless-steel backbone, carrying the connection to the PAL sampler, the coating and an arrow-shaped tip for septum penetration. SPME Arrow combines the benefits of conventional SPME with the larger sorption phase volumes that are used in stir bar sorptive extraction (SBSE). At the same time, the disadvantages of both techniques include the difficulty in automation for SBSE, and the small volume of sorption phase, as well as the low robustness of classical SPME fibers. The results indicated that extraction efficiency significantly benefited from the larger sorption phase volume. 

### 2.4. Stir Bar Sorptive Extraction (SBSE) and Stir Rod Sorptive Extraction (SRSE) of PAHs from Environmental Matrices.

Stir bar sorptive extraction was initially introduced by Baltussen et al. in 1999 [136]. In SBSE, a coated stir bar is placed into the vial together with the aqueous sample solution. Extraction of the target analytes takes place under rigorous stirring. When equilibrium is reached, the stir bar is removed and elution of the adsorbed analytes takes place either by the addition of an organic solvent or thermally [18]. SBSE by nature is an equilibrium technique, and for water samples the extraction of the target analytes into the extraction medium is controlled by the partitioning coefficient of the solutes between the coating phase and the aqueous phase [137]. Polydimethylsiloxane (PDMS) is the most used commercially available coating phase for stir bars, however, the synthesis and application of many novel coating materials has been reported.

PDMS coated SBSE bars have been successfully used for the extraction of PAHs resulting in good recoveries and low detection limits [138,139,140,141,142]. Apart from the conventional PDMS stir bars, various novel coating materials have been evaluated for the extraction of PAHs from environmental samples. Typical examples are polymeric materials that have been evaluated, as stir bar coatings are polypyrrole and polyaniline copolymer (PPy-PAN) [143] and (octyl methacrylate- ethylene dimethacrylate) copolymer [144]. Poly (ethylene glycol)-grafted multi-walled carbon nanotubes have been also evaluated for the extraction of PAHs from environmental samples [145]. In this case, the extraction efficiency was favored by the superior characteristics of MWCNTs and the ease in operation of the SBSE technique.

Hu et al. [146] prepared polydimethylsiloxane/metal–organic framework (PDMS/Al-MIL-53-NH_2_) coated stir bars and used them for the extraction of PAHs. The novel PDMS/MOFs-coated stir bars achieved higher extraction efficiencies for PAHs than the commercial PDMS-coated stir bar. In this case, the reported MOF was prepared from terephthalic acid and aluminum chloride. In contrast to the conventional PDMS coating, in which extraction is based only on hydrophobic interactions, the PDMS/MOF composite also takes advantage of the π−π conjugations.

Benede et al. [147] used stir bar dispersive liquid microextraction (SBDLME) for the extraction of PAHs from natural waters. SBDLME is a hybrid approach that combines the benefits of SBSE and DLLME. For this purpose, a neodymium stir bar was magnetically coated with a magnetic ionic liquid. At high stirring rates, the ionic liquid was dispersed into the solution, followed by being magnetically retrieved onto the stir bar when the extraction was completed. Subsequently, thermal desorption took place and PAHs were determined in a GC-MS system. The novel method exhibited high sensitivity and low LOD values.

Luo et al. [148] developed a stir rod sorptive extraction (SRSE) method for the preconcentration PAHs from water samples, prior to their determination by GC-MS. SRSE is an improved format of SBSE, which has been proposed to avoid the friction loss of extraction coatings that exist in conventional SBSE procedure. In SBSE, a stir rod (glass insert with coating) is immersed in the sample solution to adsorb the target analytes [149]. For the extraction of PAHs, a graphene-polymer composite was evaluated. Due to the high specific surface area and π–stacking properties of the hybrid coating, good extraction efficiency was reported [148].

Automated stir plate sorptive extraction (SPSE) coupled with HPLC-FLD has been also evaluated for the extraction of PAHs. For this purpose, automatic extraction, desorption and sample loading, was controlled by a programmable flow injection system, and extraction of PAHs took place on the surface of a PDMS/β-cyclodextrin/divinylbenzene (PDMS/β-CD/DVB) coated plates. The researchers investigated three different operation modes, including static, circular flow and continuous flow SPSE. It was found that extraction efficiencies with continuous flow SPSE were slightly better than circular and manual SBSE, probably due to the continuous introduction of new sample solutions [150].

### 2.5. Liquid-Phase Microextraction of PAHs from Environmental Matrices.

Liquid-phase microextraction (LPME) is a miniaturized version of classical LLE, which is characterized by minimum consumption of solvents. LPME can be divided into three main categories, the single-drop microextraction (SDME), the hollow fiber liquid-phase microextraction (HF-LPME) and the dispersive liquid-liquid microextraction (DLLME), with the latter being the most widely used LPME form [151].

HF-LPME is usually based on the use of disposable propylene porous hollow fibers that are filled with a small amount of extracting solvent (acceptor phase). In order to extract the target analytes, the fibers are immersed into the aqueous sample solution (donor phase) [151,152].

On the other hand, in single-drop microextraction (SDME), a drop acts as the acceptor phase for the extraction. SDME can be divided into two main categories i.e., the direct-immersion single-drop microextraction (DI-SDME), in which the drop is directly immersed into the sample, and the headspace single-drop microextraction (HS-SDME), in which the drop is suspending over the sample [153].

DLLME is based on the initial fast injection of a suitable mixture of two solvents, an extraction solvent and a dispersive solvent, into an aqueous sample solution with the assistance of a syringe, followed by the formation of a cloudy solution that contains droplets of the extraction solvent dispersed into the sample. After phase separation due to the difference in density of the two phases (e.g., by centrifugation), the extraction phase can be removed and analyzed. In the conventional form of DLLME, the extraction phase is accumulated at the bottom of the extraction container [151,154]. DLLME is considered to be simple, cheap and environmentally friendly, while it provides high enrichment. The proper selection of the extraction and dispersive solvents are two critical factors for the optimization of DLLME procedure. Therefore, the dispersive solvent has to be immiscible with the extraction solvent and the aqueous sample, in order to generate a cloudy solution that increases the interaction between the two phases, in order to increase the extraction efficiency [155].

Until today, many different DLLME methods have been proposed for the extraction of PAHs. In 2006, Rezaee et al. [156] developed a DLLME method for the determination of PAHs in surface water, using tetrachloroethylene and acetone as extraction and disperser solvent, respectively. In order to decrease the extraction time, the main drawback of DLLME is the requirement for organic solvents with a density higher than water, in order to be sedimented at the centrifugation step. Guo et al. [157] proposed a low-density solvent-based solvent demulsification DLLME approach. For this purpose, after the formation of the emulsion of the aqueous sample, the disperser solvent (acetone) and the extraction solvent (*n*-hexane), more acetone was added as demulsification solvent, to break up the emulsion. Since no centrifugation was required, the whole procedure was very rapid (2–3 min). A special extraction cell has been also employed for the DLLME of PAHs, based on the use of low-density solvent. Hosseini et al. [158] evaluated the application of air flotation to assist the phase separation, using toluene as an extraction solvent. In this case, the organic solvent was collected at the top part of the designed cell, and no centrifugation was required in this procedure.

The combination of ultra-sound radiation [159] and vortex radiation [160] with DLLME has been evaluated in order to assist the extraction process, and to achieve high extraction efficiency in a short period of time [161]. With the assistance of radiation, the mass transfer of target analytes from the sample to the extraction solvent was facilitated, due to the shorter diffusion distance and larger interfacial area [160].

Ultrasound-assisted emulsification microextraction (USAEME) is a similar approach with ultra-sound assisted DLLME, however, in USUAEME, the dispersion of the extraction solvent in the aqueous sample is attributed to ultra-sound radiation, and no disperser solvent is added. In this case, ultrasonic radiation is used to overcome the drawbacks of disperser solvent, such as the decrease of partition coefficient of analytes into an extraction solvent al [161,162]. USAEME methods for the extraction of PAHs from environmental samples, using toluene [161], chloroform [162], iso-octane [163] and cyclohexane [164] as extraction solvents have been reported. The combination of ultrasound radiation and iso-octane as extraction solvent enabled the handling of high volumes of sample, thus, after concentrating the organic solvent phase, enhancement factors of up to 100,000 were reported. Moreover, the phase separation of iso-octane and sample was assisted by the addition of sodium chloride (NaCl), and no centrifugation was required [163].

An interesting approach to DLLME and USAEME that can be employed for the separation of the extraction solvent is the solidification of floating organic droplet (SFO). In this case, the sample solution is transferred into an ice bath after the extraction process and the floating organic droplet is allowed to solidify. When solidified, the droplet is removed and placed in a vessel to melt prior to the instrumental analysis [165,166]. Yousefi et al. [167] evaluated the use of a deep eutectic solvent (DES) tetra-n-butyl ammonium bromide (TBAB) carboxylic acids for the extraction of PAHs from water samples based on solidification of floating organic droplet. DESs are eco-friendly solvents, which are usually prepared from a hydrogen bond acceptor (e.g., choline chloride salt) and a hydrogen bond donor (e.g., urea, glycerol, carboxylic acids etc.) that can be associated with each other with hydrogen bond interactions. Among the benefits of DESs is their easy preparation from biocompatible, nontoxic and biodegradable chemical compounds [167,168].

The application of ionic liquids in DLLME has been also studied in order to replace the environmentally hazardous volatile organic solvents that are commonly used. Pena et al. [169] developed a DLLME procedure using 1-octyl-3-methylimidazolium hexafluorophosphate ([C_8_ MiM][PF_6_ ]) IL as extraction solvent. Due to the chemical affinity between the IL and the PAHs, good extraction recoveries and enhancement factors was reported.

In order to enhance the extraction efficiency and the enhancement factors, Liu et al. [170] combined an SPE method based on a macrocyclic polyamine-functionalized silica with an ionic liquid DLLME procedure. For this purpose, after the elution of PAHs from the SPE column with acetone, distilled water and 1,3-dibutylimidazolium bis[(trifluoromethyl)sulfonyl]imide ([BBIM][Tf_2_N]) as extraction solvents were rapidly injected for the DLLME procedure. 

In order to enhance the sensitivity of the determination of PAHs in environmental matrices, Shamsipur and Hashemi [171] combined the SBSE with dispersive liquid–liquid microextraction, based on the solidification of floating organic drop. For this purpose, the target analytes were extracted on the surface of a PDMS coated stir bar, which was further placed in a glass vial containing methanol (disperser solvent). After the elution of PAHs, the stir bar was removed and 1-undecanol was added as an extraction solvent. After centrifugation and cooling in an ice bath, the organic drop was collected. Finally, the drop was again melted at room temperature, mixed with acetonitrile and analyzed in a HPLC-UV system. The combination of the extraction techniques provided good extraction recoveries and low LOD values.

Finally, Fernandez et al. [172] developed a lab on valve DLLME method for the extraction of PAHs, prior to their determination by HPLC-FLD. For this purpose, trichloroethylene was used as the extraction solvent, and acetonitrile was used as the dispersive solvent. The automated instrumentation simplified the extraction process and exhibited satisfactory enhancement factors (86–95). Table 2 summarizes the applications of DLLME and USAEME in the extraction of PAHs from water samples. Even though DLLME is the predominant form of LPME that has been employed for the extraction of PAHs, the use of SDME [173,174] and HF-LPME [175,176] has been also evaluated.

### 2.6. Fabric Phase Sorptive Extraction of PAHs from Environmental Matrices.

Fabric phase sorptive extraction (FPSE) is a novel sample preparation technique proposed by Kabir and Furton in 2014. FPSE utilizes a natural or synthetic fabric substrate, which is chemically coated in the form of ultra-thin coating with sol-gel organic-inorganic hybrid sorbent as the extraction medium. For the FPSE procedure, the sol-gel sorbent coated FPSE media is immersed into a mixture of appropriate solvents, to remove any undesirable impurities from the material, and rinsed with deionized water to remove residual organic solvents. Subsequently, the FPSE media is submerged into the sample solution placed in a glass vial. A magnet is added into the sample solution, and the sample is magnetically stirred for certain time span for the adsorption of the target analytes. Finally, the FPSE media is removed, and elution of the analytes takes place into another vial containing appropriate elution solvent. Analysis of the eluent can take place after centrifugation and or/filtration [177,178,179,180].

FPSE provides high primary contact surface area, thus, rapid and efficient analyte extraction can be easily achieved. Moreover, FPSE is also characterized by low organic solvent consumption, ease in operation, reusability and good selectivity towards the target analytes, which is based directly on the different nature of the fabric substrates and the sol-gel coating. Until today, the use of various fabric substrates, including cellulose, fiber glass and polyester, as well as several sol-gel coatings, including polyethylene glycol, polytetrahydrofuran and polydimethyldiphenylsiloxane have been evaluated as adsorbents for a wide variety of analytes [17,178,181,182].

A trace-level determination of selected PAHs in environmental water samples using FPSE prior to their determination by HPLC-FLD has been reported. For this purpose, a non-polar sol-gel C_18_ coated FPSE media was prepared and conditioned in a mixture of methanol and acetonitrile for 5 min, and then rinsed with deionized water. The extraction of PAHs took place in a glass vial containing 10 mL of the aqueous sample solution, in which the sol-gel C_18_ coated FPSE media was directly immersed. After 30 min under constant stirring at 1000 rpm, the FPSE media was removed and PAHs were eluted with acetonitrile under ultrasonic radiation for 5 min. The developed FPSE-HPLC-FLD protocol was proved to simple, efficient, fast, sensitive, green, economical and reliable for trace level determination of environmentally important PAHs [183].

Recently, fabric-phase sorptive extraction was coupled with ion mobility spectrometry (IMS) for on-site rapid detection of PAHs in aquatic environment [184]. Ion mobility spectrometry is a rapid and sensitive gas-phase analytical technique, which can be employed for the in the field testing of various chemical compounds, due to its fast analysis and compact size [185]. For the fabrication of the FPSE media, PDMS was coated on the glass fiber cloth through a sol-gel reaction. Glass wool was chosen based on the inlet temperature of IMS, since the thermal desorption of the PAHs was performed after inserting the FPSE media the inlet port of the IMS instrument directly after analyte extraction. Under optimum conditions, low LODs and satisfactory recoveries were obtaining, thus enabling the on-site monitoring water quality.

### 2.7. Other Extraction Techniques for the Determination of PAHs in Environmental Matrices.

Yang et al. [186] synthesized MOF-5 from terephthalic acid and zinc acetate, and evaluated it as a sorbent for the SPE of PAHs from environmental samples. Therefore, 300 mg of MOF-5 was packed into SPE cartridges. Although MOF-5 is known to be unstable when exposed to water, the researchers reported that the derived material of MOF-5 still demonstrated good extraction characteristics. This was attributed to the π–π conjugate effect between the terephthalic acid molecules of the framework and the PAHs and the π-complexation between PAHs and the central zinc ions. The analytes were further separated and detected in a HPLC-UV system. The method exhibited satisfactory extraction ability and low LODs (0.4–4.0 ng L^−1^), however, no sorbent reusability was reported.

Hu et al. [187] evaluated two zeolitic imidazolate frameworks for the SPE disk extraction of PAHs in aid of filter membrane of PAHs from environmental water samples. The studied ZIFs were both composed of the same metal ion (zinc) and organic linker (benzimidazole), thus differing in spatial structures with one in cube (ZIF-7), while the other was in rhombic dodecahedron (ZIF-11). ZIF-11 with markedly better extraction efficiencies due to its unique spatial structure with large cages and its molecular composition that was composed of abundant benzyl groups and metal sites on the surface.

ZIF-8 [188] has been also evaluated for the porous membrane-protected micro-solid-phase extraction (μ-SPE) of PAHs. For this purpose, the sorbent was packed in a sealed porous polypropylene membrane envelope. The novel extraction devices exhibited good extraction characteristics and decreased consumption of the organic solvent.

A molecularly imprinted polymer has been also applied for the SPE of 16 PAHs from seawater, prior to their determination by GC-MS [189]. MIPs were prepared by using the 16 PAHs mixture through non-covalent polymerization as a template based on sol-gel surface imprinting. The developed sorbent exhibited good affinity towards the target analytes. Other examples of novel SPE sorbents that have been implemented for the extraction and preconcentration of PAHs from environmental samples include a cyclodextrin-silica microporous composite [190,191], aminopropyl imidazole-modified silica gel [192], tetraazacalix[2]arene[2]triazine [193], titanium oxide nanotubes [194] and a titanate nanotube array modified by cetyltrimethylammonium bromide [195].

Krupadam et al. [196] prepared MIP microspheres in a continuous segmented flow microfluidic reactor, and used them as packing material for microtraps for the selective extraction of benzo[a]pyrene from environmental water samples. For this purpose, the pumping of monodisperse droplets of acetonitrile containing methacrylic acid as the functional monomer took place, benzo[a]pyrene was used as a template, and ethylene glycol dimethacrylate as cross-linking monomer into the microchannels of the microfluidic reactor. The obtained microspheres exhibited high extraction efficiency and selectivity towards benzo[a]pyrene. In comparison with commercially available activated carbon, the novel microspheres showed 300% higher adsorption capacity.

A portable system for the in situ extraction of PAHs was proposed by Foan et al. [197]. The researchers designed a microfluidic device for the fast extraction of PAHs using low volume samples. The work was performed on a lab-on-a-chip, made of a silicon/glass microfluidic device functionalized with PDMS. Among the benefits of the novel technique was the low organic solvent consumption and the portability. A comparison of the novel device with SBSE showed approximately 50 times less sample preparation time for the high molecular weight PAHs. However, for the lightest PAHs, the performance of the microchip required improvement.

Flow injection solid-phase extraction (FI-SPE) of PAHs from environmental samples with novel extraction sorbents has been also proposed by the research groups of Wu [198] and Zhou [199]. The first group used a multi-walled carbon nanotubes (MWCNTs)-packed micro-column for the extraction of PAHs, prior to their determination by GC-MS, while the second group prepared a copper(II) isonicotinate coordination polymer packed in a pre-column for the extraction of PAHs, prior to their determination by HPLC-DAD. Both methods exhibited good extraction characteristics. In the case of GC-MS detection, after the FI-SPE process, the eluates were collected, and manual injection was performed, while for the HPLC-DAD analysis elution of the adsorbed analytes was also performed on-line in the backflush mode by the HPLC mobile phase directly into the chromatographic column, thus minimizing the required analysis steps.

In-syringe solid-phase extraction of PAHs has been also proposed for the on-site sampling of water samples. In-syringe SPE is characterized by portability, simplicity in use, low cost and short extraction time. Zhang et al. [200] evaluated the application of MIL-101 as a novel sorbent, due to its good thermal and mechanical stability, as well as its resistance towards organic solvent and waters. The proposed technique exhibited excellent adsorption performance, since the analytes could be completely adsorbed during one adsorption cycle, thus reducing the extraction time. Moreover, it was found that the adsorbed analytes remained stable on the in-syringe device for at least 7 days.

## 3. Conclusions

A wide variety of novel microextraction techniques and miniaturized extraction techniques have been developed and applied for the extraction of PAHs from environmental samples. Most of the novel extraction techniques are variations of the conventional SPE approach, however LLE based extraction approaches have been also developed.

Unequivocally, a lot of progress has been made in the field of sorbent development for micro and miniaturized solid-phase extraction sample preparation techniques. Metal-organic frameworks, covalent organic frameworks, zeolitic imidazole frameworks, graphene, graphene oxide and carbon nanotubes are some typical examples of novel extraction sorbents that have been implemented as such, or after surface modification for the extraction of PAHs from environmental samples. At the same time, ionic liquids, polymeric ionic liquids and deep eutectic solvents have been implemented in liquid phase microextraction approaches as extraction solvents and in solid-phase extraction approaches for the surface modification of a plethora of adsorbents.

The selection of the extraction technique, as well as the extraction sorbent can be based on the needs of the analysis (e.g., selectivity) and the laboratory equipment. MSPE and d-SPE are some examples of simple, rapid and environmentally friendly extraction procedures, that have recently gained a lot of attention, due to their convenience in sample handling. The application of these techniques has rapidly increased, and a wide variety of sorbents have been evaluated. Other novel miniaturized extraction forms, including SBSE, PT-SPE and FPSE have also become popular during the last years. These techniques have been evaluated in less extend compared to SPE, MSPE and d-SPE methods, however, due to their ease in operation, they are considered useful alternatives that enrich the toolbox of analytical chemists.

Proper attention should be also given to the stability of the sorbent in the case of solid-phase extraction, since physical and chemical stability in aqueous solutions is important to provide sorbent reusability, which significantly reduces the cost of the analysis. An important technical problem that has to be overcome is the limited stability of some SPE sorbents (e.g., MOFs) in an aqueous environment that limits their applications, their potential regeneration and recyclability. Another bottleneck of many sample preparation techniques is the lack of compatibility with desorption chambers for HPLC and GC applications.

Therefore, when designing novel sorbents for the extraction of PAHs from environmental matrices, their stability should be carefully examined. Moreover, it is generally preferred to prepare sorbents rich in π-electron moieties. In this case, the extraction of PAHs can be assisted through π-stacking, as well as hydrophobic and hydrogen interactions. Additionally, developing materials with an improved affinity towards PAHs, such as MIPs, has also proved to enhance the selectivity and sensitivity of PAHs extraction. Finally, sorbent functionalization with appropriate materials, such as polymers, ionic liquids etc., can also be beneficial for the overall extraction process.

Coupling and combination of different extraction approaches has been also reported for the efficient extraction and enhancement of PAHs from environmental matrices. For this purpose, a solid-phase extraction technique (e.g., SBSE, d-SPE) can be followed by an LPME technique, to further increase the extraction sensitivity.

Future perspectives in the field of sample preparation of environmental samples for PAHs extraction, should focus on developing robust extraction approaches of the on-site extraction and the determination of the environmental contaminants. For this kind of approach, several sorbents and extraction techniques can be examined in order to develop accurate, sensitive and selective sample preparation methods.

## Figures and Tables

**Table 1:** Application of different sorbents for the magnetic solid-phase extraction (MSPE) of PAHs from environmental samples.

Sorbent	Matrix	Analytical Technique	Sorbent Mass (mg)	Time (min)	LODs (ng L^−1^)	Extraction Recovery (%)	Reusability	Ref.
HKUST-1	Water	UHPLC-FLD	5 Fe_3_O_4_/20 HKUST-1	10	0.8–12	39–59	NA	[59]
MIL-101(Cr)	Water	HPLC-PDA	1 Fe_3_O_4_@SiO_2_/0.6 MIL-101	20	2.8–27.2	NA	NA	[60]
Fe@MIL-101(Cr)	Water	HPLC-DAD	50	50	44–64	>80	At least 10 times	[61]
MIL-100(Fe)	Water	HPLC-FLD	10	10	32–2110	>80	NA	[62]
MIL-100(Fe)	Water	GC-FID	12.5	15	4.6–8.9	73–96	Up to 10 times	[63]
Fe_3_O_4_@ polydopamine/ZIF-7	Water, particulate matter	GC-MS	3 Fe_3_O_4_@PDA 15 ZIF-7	40	0.71–5.79	>82	At least 10 times	[64]
TpPa-1 COF	Water	HPLC-FLD	5	21	0.24–1.01	73–110	NA	[65]
COF-LZU1@PEI@Fe_3_O_4_	Water, soil	HPLC-FLD	5	33	0.2–20	NA	At least 6 times	[66]
G/CNF	Water	GC-FID	20	10	4–30	63.0–84.5	Up to 6 times	[67]
Fe_3_O_4_/C	Water	HPLC-FLD	50	30	0.2–0.6	76–110	At least 10 times	[68]
Hydrophilic Fe_3_O_4_/C	Water	GC-MS	10	30	15–335	NA	NA	[5]
CNF	Water	GC-FID	10	12	8–30	NA	At least 10 times	[7]
G/Fe_3_O_4_@PT	Water	GC-FID	20	10	9–20	83–107	At least 17 times	[69]
GO	Water	HPLC-UV	40	16	90–190	76.8–103.2	NA	[6]
GO-Fe_3_O_4_@PS	Water	GC-FID	15	10	3–10	69.5–88.7	NA	[70]
Poly(Py-co-Ani)@GO-Fe_3_O_4_	Water	GC-FID	35		3–10	50.4‒78.3	At least 20 times	[71]
CNTs	Water	UHPLC-FLD	5	10	25–73	76.4–106.5	Up to 3 times	[72]
mag-MIP	Water	HPLC-PDA	20	55	1.3–969	46–100	At least 3 times	[73]
mag-MIP	Water	GC-MS	5–20	17	30–750	>76	NA	[74]
RAFT-MIP	Water	GC-MS	10	9	1–100	4.5–97	NA	[8]
PDA	Water	HPLC-FLD	20	5	0.5–1.9	76.4–107	NA	[75]
PPy	Water	GC-MS	20	3	0.38–5.01	27.4- 115.7	NA	[76]
PANI/Alginate	Water	HPLC-FLD	400	20	10	86.0–97.8	Up to 6 times	[77]
PoT	Water	GC-FID	60	15	0.3–5.5	NA	Up to 15 times	[58]
IL-MNPs	Water	GC-MS	30	8	40–1111	75–102	Up to 10 times	[78]
MNP@CN/IL	Leachate, sludge	HPLC-DAD	30	35	400–590	89.50–110.2	NA	[79]
MNP-PANI-DICAT	Water, sludge, soil	GC-MS	15	40	0.8–208.6	80.2–111.9	Up to 5 times	[80]
Fe_3_O_4_@IL@MO	Water	HPLC-FLD	18	26	0.1–2	NA	NA	[81]
Fe_3_O_4_@SiO_2_@Nap	Water	HPLC-FLD	40	12	0.04–0.12	>90	At least 10 times	[1]
PC	Water, milk	HPLC-FLD	100	10	0.2–0.6	>90	NA	[82]
Fe_3_O_4_-DVB-SO_3_-	Water	GC-MS	50	10	0.6–2.1	79.9–115.3	NA	[83]
MPNP	Water	UHPLC-DAD	200	15	10.83–18.53 nM	75.7–106.4	At least 5 times	[3]
Fe_3_O_4_/SiO_2_/TPA	Water	HPLC-FLD	50	15	0.04–37.5	NA	NA	[84]
C_18_	Water	GC-MS	50	6	0.8–36 × 103	35–99	NA	[85]
C_10_–C_18_ carboxylates	Water	HPLC-FLD	200	18	0.1–0.25	>90	Up to 5 times	[86]
n-octadecylphosphonic acid	Water	GC-MS	50	1	14.1–70.0 × 103	61.9–119.1	NA	[87]
Nylon 6	Water	HPLC-PDA	40	30	0.05–0.58 × 103	36.2–87.0	NA	[88]
CTAB	Water	UHPLC-FLD	100 Fe_3_O_4_/50 CTAB	30	0.4–10.3	59.23–87.95	NA	[89]
Palm fatty acid	Leachate, sludge	HPLC-DAD	15	25	10–50	>81.1	Up to 5 times	[90]
TBCD	Water	HPLC-FLD	80	15	0.03–1.2	>80	NA	[91]
TCT	Water, urine	HPLC-FLD	40	13	0.09–0.15	89–93	At least 30 times	[92]
C_16_-HO	Water	HPLC-UV	30	24	0.14–0.31	88–95	Up to 4 times	[93]

**Table 2:** Applications of dispersive liquid-liquid microextraction (DLLME) and ultrasound-assisted emulsification microextraction (USAEME) in the extraction of PAHs from water samples.

Matrix	Analytical Technique	Extraction Solvent	Disperser Solvent	Phase Separation	LODs(ng·L^−1^)	EF	Extraction Recovery (%)	Ref.
Surface water	GC-MS	Tetrachloroethylene	Acetone	Centrifugation	7–30	603–1113	-	[156]
Rainwater	GC-MS	*n*-Hexane	Acetone	Addition of demulsification solvent	3.7–39.1	NA	-	[157]
River water	GC-FID	Toluene	Methanol	Air flotation	14–41 × 10^3^	NA	-	[158]
Sea water	GC-MS	Tetrachloroethylene	Diethyl Ether	Centrifugation	1–10	722–8133	59.2–90.5	[159]
Sediment	HPLC-FLD	Dichloromethane	Acetonitrile	Centrifugation	2.3–6.8 ng g^−1^	NA	>94.0	[160]
Tap, sea and spring water	GC-FID	Toluene	-	Centrifugation	20–50	1776–2714	99–103	[161]
Tap, well, surface water etc.	GC-MS	Chloroform	-	Centrifugation	1–36	NA	-	[162]
Tap, spring, surface water etc.	GC-MS	Iso-octane	-	Addition of NaCl	0.001–0.009	Up to 100000	-	[163]
Tap, rain and wastewater	HPLC-FLD	Cyclohexane	-	Centrifugation	0.6–62.5	90–247	95–100	[164]
Well, river, lake water etc.	HPLC-FLD	TBAB/2-decanoic acid DES	-	Centrifugation/Solidification	0.7–6.6	163–198	>80.0	[167]
Tap, bottle, fountain water etc.	HPLC-FLD	[C_8_ MiM][PF_6_ ]	Acetone	Centrifugation	0.03–2	301–346	-	[169]
Tap, well, surface water etc.	HPLC-UV	[BBIM][Tf_2_N]	Acetone	Centrifugation	2	2768–5409	-	[170]
Tap, rain and surface water	HPLC-FLD	Trichloroethylene	Acetonitrile	-	20–600	86–95	-	[172]
